# Analyses of non-benzodiazepine-induced adverse events and prognosis in elderly patients based on the Japanese adverse drug event report database

**DOI:** 10.1186/s40780-018-0106-2

**Published:** 2018-05-07

**Authors:** Yoshihiro Noguchi, Anri Ueno, Hayato Katsuno, Manami Otsubo, Aki Yoshida, Yuta Kanematsu, Ikuto Sugita, Tomoya Tachi, Teruo Tsuchiya, Hitomi Teramachi

**Affiliations:** 1 Laboratory of Clinical Pharmacy, Gifu Pharmaceutical University, 1-25-4, Daigakunishi, Gifu, 501-1196 Japan; 2 Community Health Support and Research Center, 5-6-1, kikuchichou, Gifu, 501-6242 Japan; 3 Laboratory of Community Healthcare Pharmacy, Gifu Pharmaceutical University, 1-25-4, Daigakunishi, Gifu, 501-1196 Japan

**Keywords:** Japanese adverse drug event report database (JADER), Signal detection, Elderly patients, Non-benzodiazepine (Z-drug)

## Abstract

**Background:**

The contents of the guidelines for the use of non-benzodiazepines (Z-drugs) differ slightly between THE JAPANESE SOCIETY OF SLEEP RESEARCH and THE JAPAN GERIATRIC SOCIETY, and the recommended directions are conflicting. Therefore, we analyzed the use of the Japanese Adverse Drug Event Report database (JADER) for identifying adverse events (AEs) caused by Z-drugs and clarifying their occurrence trend and prognosis.

**Methods:**

The signal value for comparison was calculated by using the proportional reporting ratio (PRR) and chi-squared test (χ^2^) results of data of elderly and non-elderly patients. Among AEs for which signals were detected in the elderly, we determined that those with lower signal values for non-elderly patients that were half the signal value of the elderly should be used with particular caution in the elderly. We also compared the prognoses.

**Results:**

The AEs with > 1 risk ratio (RR) in elderly and non-elderly patients were regarded as those that should be noted in the prognosis of AEs in elderly patients. Furthermore, 28 AEs were detected in elderly patients’ signals. In this study, in addition to movement disorders such as “falls” and “bone fractures,” identified by two academic societies, signal characteristics of the elderly were obtained for psychiatric disorders and eye disorders.

**Conclusions:**

There was no difference in prognosis, but these disorders could reduce the quality of life of patients. Therefore, we consider that in prescribing appropriate drug therapy for insomnia, attention should be paid to the occurrence of the AEs caused by the Z-drugs revealed by this study and the guidelines.

## Background

Insomnia is defined as the inability to sleep or sleeplessness that lasts for at least 1 month under proper sleeping conditions, and is accompanied by intense daytime fatigue and decreased concentration, energy, drowsiness, and vigor. Recently, in Japan, 20 to 25% of adults have been reported to experience insomnia [1, 2], and its incidence is particularly high in elderly people aged above 60 years [1]. Furthermore, 4 to 6% of Japanese adults routinely use sleeping pills [2], and the major drugs widely used in clinical practice are benzodiazepines (BZ drugs) and non-benzodiazepines (Z-drugs). These drugs act on benzodiazepine receptors in the brain to exert hypnotic actions by enhancing the action of the gamma-aminobutyric acid (GABA) system, which shows inhibitory action in the central nervous system (CNS). Previously, barbiturates acting on the same GABA receptor were used, but these newer drugs are considered to be less likely to cause fatal damage due to suppression of the respiratory center than barbiturates. However, the doses of BZ drugs used in Japan are higher than those used overseas. Furthermore, there is also the problem of side effects including anxiolytic actions, motor dysfunction, muscle relaxant action, memory disorders, and long-term use dependency and tolerance.

Therefore, the “ Clinical guidelines for proper use of sleeping pills and drug withdrawal” of THE JAPANESE SOCIETY OF SLEEP RESEARCH states that the quality of evidence is “moderate” and the recommendation is “weak,” and it recommends the use of Z-drugs for elderly people who experience sleeplessness [3]. Alternatively, the “Guidelines for Medical Treatment and its Safety in the Elderly 2015” of THE JAPAN GERIATRIC SOCIETY indicate that the quality of the evidence is “moderate” and the recommendation degree is “strong.” Therefore, long-term use of Z-drug among the elderly is not recommended [3].

Thus, THE JAPANESE SOCIETY OF SLEEP RESEARCH evaluated “the presence or absence of drug use” and THE JAPAN GERIATRIC SOCIETY evaluated the “long-term use of drugs,” and although the contents of their guidelines differ, the recommended direction is contradictory [3].

These guidelines are prepared using a method similar to that of the Grading of Recommendations Assessment, Development, and Evaluation (GRADE) system. In determining the recommendation level, there is also the challenge of insufficient documented proof, because the empirical evidence is not a research topic and the evidence is scarce because of the limited data on the elderly. Therefore, repeated discussions and voting by the research groups responsible for preparing guidelines have provided some evidence, which has been included in the guidelines even if it is not adequate [3].

However, the members of the GRADE working group have pointed out that the original method of preparation used by THE JAPAN GERIATRIC SOCIETY did not follow the GRADE system. Specifically, issues were pointed out such as the fact that “evidence is not integrated with outcomes as the main body” and “the evaluation method of the quality of evidence is uncertain” [3]. THE JAPANESE SOCIETY OF SLEEP RESEARCH doubts that the recommendations of THE JAPAN GERIATRIC SOCIETY differ and strongly criticize their methods, indicating that there are problems with document searches [3].

Furthermore, the method used by THE JAPANESE SOCIETY OF SLEEP RESEARCH also points out the following problems by Okumura [3]. These problems include the following: “the collection year of the document is not up to date,” “the description of statistical significance is incomprehensible (the confidence interval includes a zero, and there is no statistical significance),” the quality of the evidence including ‘the method of evaluation is unclear’, and “no document is cited by the integrated primary research, which is not clearly specified [3].”

To use drugs appropriately, it is important to understand the adverse events (AEs) they cause. Signals based on the principle of disproportionality focusing on the difference in the number of reported cases of AEs are used as indicators of the safety of drugs. Signals are considered to be able to detect unknown AEs at an early stage, and numerous risk assessments have been reported [4–8]. These signals include proportional reporting ratio (PRR) [9] and reporting odds ratio (ROR) [10]. There are also signals such as the Bayesian Confidence Propagation Neural Network (BCPNN) [11] and Gamma-Poisson Shrinker (GPS) [12] that use Bayesian estimations. Furthermore, we have also reported the analysis of prognostic comparisons of elderly and non-elderly patients [13].

Therefore, in this study, we used the Japanese Adverse Drug Event Report (JADER) database of a spontaneous reporting system (SRS) in Japan, to identify AEs caused by Z-drugs and analyze their trends.

## Methods

### Data source

The JADER is a database of post-marketing surveillance and spontaneous reports collected by Pharmaceuticals and Medical Devices Agency (PMDA) as Japanese regulatory authority. This database is used for Japanese pharmacovigilance, and it is similar to the database for FAERS in the United States, and other countries for pharmacovigilance. The JADER database was downloaded from the website of PMDA. It consists of four datasets in csv format: patient demographic information (DEMO.csv), drug information (DRUG.csv), AE information (REAC.csv), and primary disease information (HIST.csv). In this study, data from patients registered in JADER from the first quarter of 2004 to the fourth quarter of 2015 were used. However, reports with missing information on sex, age, or primary disease, and where subjective terms such as “youth” and “elderly” were used, were excluded from the analysis data.

### Definitions of suspected drugs and AEs

The suspected drugs were the Z-drugs zolpidem, eszopiclone, and zopiclone. AE is registered in the JADER database as the preferred term (PT) of the Medical Dictionary for Regulatory Activities/Japanese version (MedDRA/J). In this study, the target AE was defined as the high-level group term (HLGT).

### Definition of elderly patients

Data on the age of patients in the cases registered in the JADER database are provided as data collected every 10 years in principle for privacy consideration. The definition of elderly and non-elderly patients has ambiguous and subjective parts, and the determination is challenging. According to the definition of the United Nations, elderly people are those over 60 years old. The World Health Organization (WHO) defines people over the age of 65 as elderly people, and in numerous countries, those 65 years and older are defined as elderly people. Therefore, in this study, similar to previous reports [8, 13, 14], we considered elderly patients as those > 60 years old and non-elderly patients as those < 60 years.

### Comparison of signal values

Signal values to be compared were calculated from the PRR and chi-squared test (χ^2^) values of elderly and non-elderly patients, respectively, using formula (1) proposed by Takagi et al. [15]:

1 $$ \mathrm{Signal}\kern0.34em \mathrm{value}=\ln \kern0.01em \left(\mathrm{PRR}\right)+\ln \kern0.01em \left({\upchi}^2\right) $$

In this study, among the AEs for which signals were detected in the elderly, we determined that those with signal values in non-elderly patients that were lower than half of that in the elderly should be used with particular caution in the elderly using formula (2):

2 $$ 1/2\;\left(\mathrm{elderly}\;{\mathrm{patients}}^{'}\;\mathrm{signal}\kern0.17em \mathrm{value}\right)\hbox{-} \mathrm{non}\hbox{-} \mathrm{elderly}\;{\mathrm{patients}}^{'}\;\mathrm{signal}\kern0.17em \mathrm{value}>0 $$

As this signal value cannot be calculated as having 0 reports of AEs, we corrected the value by adding 0.5 (Haldane–Anscombe 1/2 correction) [16].

### Prognosis of AEs

From the registered outcome information, “recovery” and “ light ” were classified as good prognosis. In contrast, “death,” “with sequelae,” and “unrecovered” were classified as AEs with poor prognoses. We created a 2 × 2 contingency table based on the elderly/non-elderly and good/bad prognosis of classified AEs and calculated the risk ratio (RR), the 95% confidence interval (CI), and χ^2^ value [13]. As 0 cells in the 2 × 2 contingency table cannot be the result of the calculation, we corrected this bias by adding 0.5 to all cells (Haldane–Anscombe 1/2 correction) [17].

## Results

We obtained data on 176,184 cases of oral drugs use by patients (elderly and non-elderly patients, 110,126 and 66,058 cases, respectively) excluding cases where information on sex and age were unclear from the analysis data table based on patient background factor. Of the 321 AEs, 28 were detected in elderly patients’ signals (Table 1). Among the 28 AEs, the following 12 were particularly determined to require cautious monitoring in the elderly: glaucoma and ocular hypertension (n_11_: 10, PRR: 21.3, χ^2^: 152.6, signal value: 8.1), impulse control disorders not elsewhere classified (NEC) (n_11_: 6, PRR: 26.0, χ^2^: 102.5, signal value: 7.9), neurological| special senses and psychiatric investigations (n_11_: 6, PRR: 13.4, χ^2^: 52.2, signal value: 6.5), injuries by physical agents (n_11_: 7, PRR: 6.4, χ^2^: 25.6, signal value: 5.1), personality disorders and disturbances in behavior (n_11_: 5, PRR: 7.6, χ^2^: 21.3, signal value: 5.1), neurological disorders of the eye (n_11_: 9, PRR: 4.6, χ^2^: 21.3, signal value: 4.6), manic and bipolar mood disorders and disturbances (n_11_: 3, PRR: 6.7, χ^2^: 9.0, signal value: 4.1), movement disorders (include parkinsonism) (n_11_: 21, PRR: 2.7, χ^2^: 19.9, signal value: 4.0), enzyme investigations NEC (n_11_: 17, PRR: 2.4, χ^2^: 12.4, signal value: 3.4), disturbances in thinking and perception (n_11_: 12, PRR: 2.5, χ^2^: 9.1, signal value: 3.1), vision disorders (n_11_: 7, PRR: 2.6, χ^2^: 5.3, signal value: 2.6), and ocular infections irritations and inflammations (n_11_: 8, PRR: 2.4, χ^2^: 5.0, signal value: 2.5).

**Table 1:** Comparison of signal values of elderly and non-elderly patients

adverse event (High Level Group Term)	elderly patients (over the age of 60)					non-elderly patients (under the age of 60)			
	n_11_	PRR	χ2	signal value	n_11_	PRR	χ2	signal value	risk Judgment
Deliria (include confusion)	84	16.2	1079.4	9.8	34	12.0	304.3	8.2	
Product use issues	14	40.0	391.1	9.7	31	23.0	536.5	9.4	
Sleep disturbances (include subtypes)	26	14.9	294.4	8.4	14	6.8	60.9	6.0	
Sleep disturbances	38	10.8	309.3	8.1	24	6.0	91.0	6.3	
Glaucoma and ocular hypertension	10	21.3	152.6	8.1	0(0.5)	0(1.6)	0.1(0.3)	-(−0.7)	×
Psychiatric and behavioural symptoms NEC	22	13.7	225.8	8.0	24	4.4	58.7	5.6	
Impulse control disorders NEC	6	26.0	102.5	7.9	0(0.5)	0(1.0)	0.001(0.5)	-(−0.7)	×
Dementia and amnestic conditions	21	10.0	152.3	7.3	13	14.4	134.5	7.6	
Suicidal and self-injurious behaviours NEC	19	10.4	142.0	7.3	49	7.3	250.17.5		
Mental impairment disorders	22	8.3	127.8	7.0	14	10.1	98.4	6.9	
Neurological disorders NEC	153	3.6	294.5	7.0	103	3.6	194.7	6.5	
Neurological special senses and psychiatric investigations	6	13.4	52.2	6.5	1	2.1	0.0007	−6.5	×
Psychiatric disorders NEC	39	4.0	83.1	5.8	50	6.1	203.1	7.1	
Respiratory disorders NEC	51	2.9	60.8	5.2	35	2.7	36.1	4.6	
Injuries by physical agents	7	6.4	25.6	5.1	2	1.2	0.0008	−4.6	×
Personality disorders and disturbances in behavior	5	7.6	21.3	5.1	3	1.9	0.6	0.1	×
Neurological disorders of the eye	9	4.6	21.3	4.6	4	2.4	2.0	1.6	×
Injuries NEC	35	2.6	33.0	4.5	17	2.6	15.4	3.7	
Changes in physical activity	6	4.9	14.7	4.3	4	2.8	3.0	2.2	
Manic and bipolar mood disorders and disturbances	3	6.7	9.0	4.1	2	1.2	0.02	−3.8	×
Movement disorders (include parkinsonism)	21	2.7	19.9	4.0	12	1.6	2.1	1.2	×
Anxiety disorders and symptoms	6	3.7	9.0	3.5	7	2.1	3.0	1.8	
Therapeutic and nontherapeutic effects (exclude toxicity)	15	2.6	12.7	3.5	16	3.0	19.1	4.1	
Enzyme investigations NEC	17	2.4	12.4	3.4	3	0.4	2.1	−0.2	×
Disturbances in thinking and perception	12	2.5	9.1	3.1	5	1.2	0.05	−2.9	×
Vision disorders	7	2.6	5.3	2.6	3	1.6	0.2	−0.9	×
Ocular infections irritations and inflammations	8	2.4	5.0	2.5	0(0.5)	0(0.1)	3.7(2.9)	-(−1.2)	×
Depressed mood disorders and disturbances	5	2.8	4.1	2.4	6	2.5	3.9	2.3	

*※ n*_*11*_ the number of co-occurrences of interest, *PRR* proportional reporting ratio, signal value: ln(PRR) + ln(χ2), risk judgment: low signal values of non-elderly patients than half of the signal value of the elderly, *NEC* not elsewhere classified

In addition, when these 12 AEs were counted using the System Organ Class (SOC), four and three HLGTs were classified as psychiatric disorders and eye disorders, respectively (Tables 2, 3).

**Table 2:** Number and adverse event of signal detection included in SOC.

adverse event: SOC (Number of HGLTs included in SOC)	adverse event: HGLT
Psychiatric disorders (13)	Deliria (include confusion)
	Sleep disturbances
	Psychiatric and behavioural symptoms NEC
	Impulse control disorders NEC
	Dementia and amnestic conditions
	Suicidal and self-injurious behaviours NEC
	Psychiatric disorders NEC
	Personality disorders and disturbances in behavior
	Changes in physical activity
	Manic and bipolar mood disorders and disturbances
	Anxiety disorders and symptoms
	Disturbances in thinking and perception
	Depressed mood disorders and disturbances
Nervous system disorders (5)	Sleep disturbances (include subtypes)
	Mental impairment disorders
	Neurological disorders NEC
	Neurological disorders of the eye
	Movement disorders (include parkinsonism)
Movement disorders (include parkinsonism)	Product use issues
	Injuries by physical agents
	Injuries NEC
Eye disorders (3)	Glaucoma and ocular hypertension
	Vision disorders
	Ocular infections irritations and inflammations
Investigations (2)	Neurological special senses and psychiatric investigations
	Enzyme investigations NEC
Respiratory thoracic and mediastinal disorders (1)	Respiratory disorders NEC
General disorders and administration site conditions (1)	Therapeutic and nontherapeutic effects (exclude toxicity)

*SOC* System Organ Class, *HLGT* High Level Group Term, *NEC* not elsewhere classified

**Table 3:** Number and adverse event of signal detection in elderly risk included in SOC.

adverse event: SOC (Number of HGLTs included in SOC)	adverse event: HGLT
Psychiatric disorders (4)	Impulse control disorders NEC
	Personality disorders and disturbances in behavior
	Manic and bipolar mood disorders and disturbances
	Disturbances in thinking and perception
Eye disorders (3)	Glaucoma and ocular hypertension
	Vision disorders
	Ocular infections irritations and inflammations
Nervous system disorders (2)	Neurological disorders of the eye
	Movement disorders (include parkinsonism)
Investigations (2)	Neurological special senses and psychiatric investigations
	Enzyme investigations NEC
Injury poisoning and procedural complications (1)	Injuries by physical agents

*SOC* System Organ Class, *HLGT* High Level Group Term, *NEC* not elsewhere classified

Among the AEs for which signals were detected in the elderly, none led to poor prognosis in the elderly compared to the non-elderly (Table 4). (Table 4-1: Especially the prognostic comparison of adverse events [AEs] characterized by the elderly. (Table 4-2: Prognostic comparison of other adverse events [AEs]).

**Table 4:** Comparison of prognosis of elderly and non-elderly patients

Adverse event (High Level Group Term)	RR (95%CI)	χ2
Especially the prognostic comparison of AEs characterized by the elderly		
Glaucoma and ocular hypertension	0.33 (0.12–0.92)	0.32
Impulse control disorders NEC	0.08 (0.01–1.10)	0.09
Neurological special senses and psychiatric investigations	1.00 (1.00–1.00)	NA
Injuries by physical agents	0.33 (0.01–12.81)	0.37
Personality disorders and disturbances in behaviour	1.91 (0.10–34.92)	0.13
Neurological disorders of the eye	0.67 (0.27–1.63)	0.04
Manic and bipolar mood disorders and disturbances	0.71 (0.02–25.32)	0.86
Movement disorders (include parkinsonism)	0.35 (0.04–3.46)	0.10
Enzyme investigations NEC	0.23 (0.02–2.73)	0.06
Disturbances in thinking and perception	0.39 (0.01–16.90)	0.42
Vision disorders	4.85 (0.35–66.36)	0.99
Ocular infections irritations and inflammations	0.47 (0.21–1.00)	0.53
Prognostic comparison of other AEs		
Deliria (include confusion)	3.91 (0.22–68.39)	0.30
Product use issues	0.67 (0.09–4.83)	0.02
Sleep disturbances (include subtypes)	1.82 (0.23–14.26)	0.004
Sleep disturbances	1.53 (0.33–7.13)	0.02
Psychiatric and behavioural symptoms NEC	2.88 (0.71–11.73)	1.55
Dementia and amnestic conditions	0.80 (0.06–11.50)	0.33
Suicidal and self-injurious behaviours NEC	0.95 (0.48–1.89)	0.02
Mental impairment disorders	0.87 (0.06–12.52)	0.40
Neurological disorders NEC	1.18 (0.47–2.91)	0.01
Psychiatric disorders NEC	0.31 (0.04–2.65)	0.46
Respiratory disorders NEC	0.48 (0.27–0.87)	5.54
Injuries NEC	1.20 (0.37–3.93)	0.01
Changes in physical activity	0.80 (0.07–9.18)	0.39
Changes in physical activity	0.80 (0.07–9.18)	0.39
Therapeutic and nontherapeutic effects (exclude toxicity)	0.44 (0.02–9.61)	0.04
Depressed mood disorders and disturbances	1.5 (0.13–17.67)	0.23

*※AEs* adverse events, *RR* risk ratio, *95%CI* 95% confidence interval, *NEC* not elsewhere classified

## Discussion

“THE JAPANESE SOCIETY OF SLEEP RESEARCH” and “THE JAPAN GERIATRIC SOCIETY” have completely different recommendations on the use of Z-drugs. The GRADE system for preparing guidelines is not a method that guarantees reproducibility but one that increases transparency.

Therefore, the recommendations proposed by independent research teams may differ because of this characteristic. However, these guidelines have become an evaluation of the quality of unclear evidence [3].

Recently, the improvement in information technology has enabled the analyses of numerous large datasets, and it is expected that accurate evidence could be obtained using patient data in clinical practice. Therefore, in this study, we comprehensively analyzed datasets from JADER to clarify the tendency of AE occurrences and prognoses of nonbenzodiazepine hypnotic drugs in elderly patients.

Among the 28 AEs identified, the following 12 were particularly determined to require cautious monitoring in the elderly: glaucoma and ocular hypertension, impulse control disorders NEC, neurological special senses and psychiatric investigations, injuries by physical agents, personality disorders and disturbances in behavior, neurological disorders of the eye, manic and bipolar mood disorders and disturbances, movement disorders (include parkinsonism), enzyme investigations NEC, disturbances in thinking and perception, vision disorders, and ocular infections irritations and inflammations. Regarding the manifestation of these AEs, medical personnel including pharmacists need to pay attention to elderly patients administered Z-drugs.

In this study, we found that following psychiatric disorders, AEs related to eye disorders were increasingly exhibited by the elderly. Among eye disorders, “blepharospasm”, which is a local dystonia is a problem recently identified as an AE of benzodiazepines and Z-drugs. Blepharospasm is considered to cause diseases that induce strong sensory hypersensitivity and lead to eyelid loss.

Benzodiazepines and Z-drugs have been found to cause side effects that interfere with comfortable vision such as robust and persistent “dazzling,” “pain,” and “blurred vision.” Wakakura et al. [17] have warned that these symptoms are likely “benzodiazepine induced blepharospasm”.

In “benzodiazepine induced blepharospasm,” a few patients are aware of the dazzling, pain, and foggy feeling. However, in general ophthalmology, this disorder may be treated as dry eye or asthenopia, and patients may be considered nervous and fixated on their eyes, although they are thought to be healthy. Therefore, it is also important for a pharmacist who has a wide knowledge of drugs to confirm these observations [18].

Analysis using spontaneous report databases such as the JADER used in this study could identify “AEs that are highly related to expression risk” and “AEs related to poor prognosis” in elderly patients. However, the spontaneous report database cannot provide the total number of patients using the target Z-drug. In addition, because the number of AEs registered does not correspond to the total number of patients who develop AEs, their incidence cannot be calculated. It should be noted that some AEs may be very rarely expressed despite their association with a higher risk of development in the elderly than in the non-elderly patients. Thus, there are certain limitations in analyzing the results of studies using data from spontaneously reporting databases, and further detailed clinical research would be required in the future. The WHO defines a safety signal as: is “Reported information on a possible causal relationship between an adverse event and a drug, the relationship being unknown or incompletely documented previously” [19], and it cannot be concluded simply by detecting a signal. However, those with large differences in signal values are characterized, and attention needs to be considered. In addition, in this study, there were some AEs with few differences, but further investigation is necessary due to future accumulation of case reports.

## Conclusions

In this study, in addition to movement disorders such as “falls” and “bone fractures” recorded by “THE JAPANESE SOCIETY OF SLEEP RESEARCH” and “THE JAPAN GERIATRIC SOCIETY,” signals that are characteristic to the elderly were obtained for psychiatric disorders and eye disorders. These disorders are possible irreversible progressions of symptoms and could reduce the quality of life of patients. However, as they are concerned about in the report, eye disorders caused by benzodiazepine receptor agonists in Japan are not recognized as important adverse events in Japan. This study is a comprehensive analysis of adverse events, and more detailed analysis is required. However, in addition to each clinical practice guideline, attention must be paid to the manifestation of AEs caused by Z-drugs revealed in this study until more detailed analysis results are obtained.

We consider that appropriate drug therapy for insomnia should be implemented by practicing side effect management. This would include considering individual patients and paying attention to the occurrence of Z-drug-induced AEs revealed in this study and the guidelines of each academic society. And we believe that this paper will contribute to future detailed side effects studies and proper use of Z-drugs.

## Abbreviations

AE: Adverse event
BCPNN: Bayesian confidence propagation neural network
BZ: Benzodiazepine
CI: Confidence interval
CNS: Central nervous system
GABA: Gamma-aminobutyric acid
GPS: Gamma-Poisson Shrinker
GRADE: Grading of recommendations assessment, development, and evaluation
HLGT: High-level group term
JADER: Japanese adverse drug event report database
MedDRA/J: Medical dictionary for regulatory activities/Japanese version
NEC: Not elsewhere classified
PRR: Proportional reporting ratio
PT: Preferred term
ROR: Reporting odds ratio
RR: Risk ratio
SOC: System organ class
SRS: Spontaneous reporting system
WHO: World Health Organization
Z-drug: Non-benzodiazepine
